# A Working Hypothesis Regarding Identical Pathomechanisms between Clinical Efficacy and Adverse Reaction of Clozapine via the Activation of Connexin43

**DOI:** 10.3390/ijms21197019

**Published:** 2020-09-24

**Authors:** Motohiro Okada, Kouji Fukuyama, Takashi Shiroyama, Masahiko Murata

**Affiliations:** 1 Department of Neuropsychiatry, Division of Neuroscience, Graduate School of Medicine, Mie University, Tsu 514-8507, Japan; k-fukuyama@clin.medic.mie-u.ac.jp (K.F.); takashi@clin.medic.mie-u.ac.jp (T.S.); 2 National Hospital Organization Sakakibara Hospital, 777 Sakakibara, Tsu, Mie 514-1292, Japan; muyuhton@gmail.com

**Keywords:** clozapine, adverse drug reaction, schizophrenia, connexin, protein kinase B

## Abstract

Clozapine (CLZ) is an approved antipsychotic agent for the medication of treatment-resistant schizophrenia but is also well known as one of the most toxic antipsychotics. Recently, the World Health Organization’s (WHO) global database (VigiBase) reported the relative lethality of severe adverse reactions of CLZ. Agranulocytosis is the most famous adverse CLZ reaction but is of lesser lethality compared with the other adverse drug reactions of CLZ. Unexpectedly, VigiBase indicated that the prevalence and relative lethality of pneumonia, cardiotoxicity, and seizures associated with CLZ were more serious than that of agranulocytosis. Therefore, haematological monitoring in CLZ patients monitoring system provided success in the prevention of lethal adverse events from CLZ-induced agranulocytosis. Hereafter, psychiatrists must amend the CLZ patients monitoring system to protect patients with treatment-resistant schizophrenia from severe adverse CLZ reactions, such as pneumonia, cardiotoxicity, and seizures, according to the clinical evidence and pathophysiology. In this review, we discuss the mechanisms of clinical efficacy and the adverse reactions of CLZ based on the accumulating pharmacodynamic findings of CLZ, including tripartite synaptic transmission, and we propose suggestions for amending the monitoring and medication of adverse CLZ reactions associated with pneumonia, cardiotoxicity, and seizures.

## 1. Introduction

More than 30% of patients on the schizophrenia spectrum do not show a response against two consecutive antipsychotic medications [1]. These patients are considered to have treatment-resistant schizophrenia [2,3]. Treatment-resistant schizophrenia is defined as not achieving a sufficient treatment response (i.e., persistent positive symptoms of at least moderate severity) by two or more antipsychotics trials from at different chemical classes at a recognized therapeutic dosage used for at least six weeks [4,5]. The guidelines of several organizations recommend that clozapine (CLZ) should be initiated in patients with treatment-resistant schizophrenia [6]. CLZ is the only approval antipsychotic agent for the treatment of treatment-resistant schizophrenia and one of the most effective antipsychotics [7], as 30–60% of patients with treatment-resistant schizophrenia were shown to respond to CLZ therapy [8,9,10]. Indeed, a recent systematic review and meta-analysis study demonstrated that CLZ, compared with other atypical antipsychotics, was associated with lower hospitalization and all-cause discontinuation rates, as well as better outcomes regarding overall symptoms [11].

In clinical practice, guidelines are not always followed and only 30% of treatment-resistant schizophrenia patients receive CLZ treatment [12]. The delay in the initiation of CLZ pharmacotherapy, defined as the time from meeting the treatment-resistant schizophrenia criteria until initiating CLZ pharmacotherapy, was found to be approximately 4–6 years [13,14,15]. Delays in the adequate treatment of treatment-resistant schizophrenia lead to negatively influencing the quality of life of patients with schizophrenia, which is clinically undesirable as increasing numbers of psychotic exacerbations impair daily and occupational functioning. The majority of psychiatrists are aware of the effectiveness of CLZ [16,17]; however, the reasons for the underutilization of CLZ concern the possible severe/lethal adverse drug reactions and rigorous clinical monitoring required [16,17,18]. In other words, many psychiatrists evaluate that the possible adverse effects of CLZ outweigh its benefits [19].

Recently, several systematic review and meta-analysis studies demonstrated that CLZ was an antipsychotic class of the highest efficacy against treatment-resistant schizophrenia with the lowest frequency of neuropsychiatric side effects [20]. An 11-year follow-up of mortality in patients with schizophrenia: a population-based cohort (FIN11) study reported that the long-term intake of antipsychotics was related to an overall lower mortality than placebo, with CLZ carrying the lowest mortality in all antipsychotics [21]. In spite of these advantages of CLZ, the Food and Drug Administration (FDA) states five Black Box warnings, including neutropenia (due to the risk of agranulocytosis), orthostatic hypotension, seizures, myocarditis, and dementia (risk of a cardiovascular event) [22]. These discrepancies in the evaluation of CLZ among medical evidence, the recognition of psychiatrists, and strict administrative regulation for CLZ are involved in its features due to the lower type A adverse reactions and higher type B adverse reactions of CLZ when compared with other antipsychotics [23].

Haematological monitoring of the white blood cell and absolute neutrophil count is mandatory in all countries for dispensing CLZ [24]. This stringent regulation for CLZ has been enforced based on historical tragedy. Just five months after its introduction into clinical use, CLZ had to be abruptly discontinued in 1975 due to agranulocytosis, with a 0.7% incidence (16/2260) and 50% relatively lethality (8/16) [25]. Over the last half century, psychiatry succeeded in drastically reducing the lethality of agranulocytosis associated with CLZ through the strict administrative regulation of CLZ. Indeed, a recent search of the World Health Organization’s (WHO) global database (VigiBase) indicated the largest number of reported agranulocytosis case numbers to WHO, but these became of lesser lethality compared with other adverse drug reactions to CLZ [26] (Table 1). Contrary to agranulocytosis, VigiBase indicated that the prevalence and relative lethality of pneumonia, cardiotoxicity, and seizures associated with CLZ are more severe than recognized by general psychiatrists (Table 1).

The large number of cases and the reduction in the relative lethality of agranulocytosis associated with CLZ demonstrates the epidemiological success of early detection and medication through the CLZ patients monitoring system. Therefore, CLZ patients monitoring systems should be amended by evidence/pathophysiology-based aspects. Unfortunately, until recently, psychiatry has not succeeded in proposing a pathophysiological hypothesis of these adverse CLZ reactions. In other words, it remained to be clarified whether the mechanisms of the efficacious and adverse effects of CLZ were modulated by the same or different regulation systems. Based on considering these above clinical and preclinical backgrounds of CLZ, in this review, we introduce recent clinical findings regarding the adverse CLZ reactions, except for agranulocytosis, and then discuss the common pathomechanisms of severe adverse CLZ reactions, seizures, cardiotoxicity, and pneumonia.

## 2. Adverse Drug Reactions of CLZ

It has been established that adverse drug reactions were classified into two categories [26]. The Type A reactions are predictable, common, and dose-dependent reactions, whereas Type B reactions are unpredictable, relatively rare, and idiosyncratic reactions [26]. In this review, we would like to discuss the Type B adverse drug reactions of CLZ, including pneumonia, cardiotoxicity, and seizures, which have higher relative lethality than that of agranulocytosis in association with CLZ (Table 1).

### 2.1. Seizure

The seizure risk during CLZ treatment has been estimated at roughly 1–7.5% [27,28,29]. A VigiBase search indicated that the relative lethality associated with seizures from CLZ (5%: 308 fatal outcomes in 6231 cases) was approximately 2.5 times higher than that associated with agranulocytosis (2%: 550 fatal outcomes in 34,931 cases) [23]. Tonic-clonic generalized seizures were the most reported in CLZ-induced seizure phenotypes, but other seizure types, including myoclonic, absence, simple, and complex partial seizures, have also been reported [27,30].

The regression model analysis could not detect a relationship between the dosage of CLZ and seizure occurrence; however, the analysis of variance model detected that the risk of seizure was increased in a daily CLZ dosage dependent-manner: lower than 300 mg/day (3%), 325–500 mg/day (8%), and higher than 500 mg/day (38%) [29]. Another clinical study also indicated that higher dosages (>600 mg/day) and serum concentration (>1.5 μM) were associated with seizure risk, and that exceeding 4 μM significantly increased the risk of seizures [30]. Thus, CLZ-induced seizures displayed aspects similar to dose-dependent common Type A adverse reactions.

The prevalence of electroencephalograph (EEG) abnormality in individuals with CLZ intake was surprisingly of higher incidence, ranging from 25% to 53% [31,32,33,34]. Generally, the most common EEG abnormality was nonspecific generalized slowing [32,35,36], involving increasing delta and theta waves, whereas spike/sharp activity was present in a relatively smaller proportion. Contrary to the seizure prevalence, the regression model indicated a significant relationship between the plasma CLZ levels and the prevalence of EEG abnormality.

An elevation of 0.3 μM in the plasma CLZ level increased the prevalence of EEG abnormality by 12% [27,30]. Thus, while EEG abnormalities can be asserted as a dose-dependent common Type A adverse CLZ reaction, CLZ-induced seizures require more detailed consideration. Indeed, several clinical studies of CLZ-induced seizures summarized that the occurrence of seizures was not necessarily predicted by nonspecific EEG abnormality [27,30,33,34,35,37]. Other risk factors of CLZ-induced seizures were reported. The rapid titration of the CLZ dose may produce an increased risk of seizures [27]. During the titration and initiation of CLZ intake was a high risk period of CLZ-induced seizures, whereas during the maintenance of the CLZ dosage (even after several years of therapy), CLZ-induced seizures were not rare [27,38]. Additionally, a younger age also provided a risk of seizure [27,28].

### 2.2. Cardiotoxicity

CLZ exhibited association with severe/lethal cardiotoxic adverse drug reactions, including dilated cardiomyopathy, myocarditis, and pericarditis [39,40]. VigiBase search reported relative lethality associated with myocarditis (12%: 539 fatal outcomes in 4586 cases) and cardiomyopathy (12%: 131 fatal outcomes in 1132 cases) [23] (Table.1). Until recently the prevalence of CLZ-induced cardiovascular adverse reactions has been argued as the importance of CLZ therapy. A Danish registry reported that the predicted maximum lethality associated with myocarditis was up to 0.28% (the prevalence of myocarditis and cardiomyopathy were 0.03% and 0.12%, respectively) [41]. In contrast, another study argued that with adequate monitoring of CLZ-induced cardiomyopathy/myocarditis, 3% of CLZ intake patients already exhibited cardiomyopathy/myocarditis, and those who cannot identify an incidence of 3% among their patients are ignoring numerous cases [42]. A recent systematic review and meta-analysis study reported that the significant event rates of CLZ-induced adverse drug reactions between Australia and outside of Australia were 2% and 0.3%, respectively [43]. These discrepancies among registries are likely caused by the lack of clear diagnostic criteria for CLZ-induced cardiotoxicity [44]. Although guidelines were proposed, these are not in line with current cardiac practice, and have a significant risk of either under- or over-diagnosis [44].

#### 2.2.1. Myocarditis

CLZ-induced myocarditis typically occurs during the early exposure period. The common symptoms consist of fever, chest pain, breathlessness, and palpitations [45,46]. It is typically for many patients to exhibits myocarditis of a mild grade, and be unaware until several years of exposure later, if at all. Therefore, the term “asymptomatic/atypical myocarditis” is found in the literature with regard to CLZ medication. The symptoms are varied and can even be absent unless the patient is in cardiogenic shock. Indeed, 50% of CLZ-induced asymptomatic myocarditis were described as fatal [47,48]. Rapid dose titration during the early exposure stage, concomitant with valproate and selective serotonin reuptake inhibitor (SSRI) of CLZ, increased the risk of development of myocarditis [49,50].

An electrocardiogram (ECG) is not recommended for the diagnosis of myocarditis [44,47,51], as ECG results are typically normal; however, it is not uncommon to display sinus tachycardia, nonspecific abnormalities, or specific abnormality similar to acute myocardial infarction [44,47]. Troponin has guideline-based measurements for the identification of coronary ischaemia and inflammation as an established cardiac-specific biomarker, whereas creatine kinase and c-reactive protein are not used to diagnose myocarditis due to their insufficient specificity and sensitivity [49,50].

#### 2.2.2. Cardiomyopathy

Generally, cardiomyopathy is considered to be a chronic disease, and typically manifests after months or years of CLZ exposure. Common cardiomyopathy symptoms are peripheral oedema, decreased exercise tolerance, and central congestion (raised jugular venous pressure and coarse crackles at lung bases). CLZ-induced cardiomyopathy also commonly exhibits increased breathlessness, alongside orthopnoea (the inability to lie flat), paroxysmal nocturnal dyspnoea (waking up in the night gasping for breath and needing to sit up), and increased peripheral oedema [52]. Pump failure is an established mode of death for all causes of cardiomyopathy. Contrary to myocarditis, specific risk factors associated with CLZ-induced cardiomyopathy were not identified [49].

ECG does not comprise diagnostic criteria for cardiomyopathy, but can indicate important information regarding the aetiology (Q waves seen in myocardial infarction, left ventricular hypertrophy and strain seen in hypertension, etc.) and potential therapy (QRS duration when considering the implantation of cardiac resynchronisation therapy devices) [44]. There is no specific biomarker of cardiomyopathy associated with CLZ, but elevation of the brain natriuretic peptide or N-terminal pro brain natriuretic peptide, which suggest the possibility of the existence of active myocarditis or cardiomyopathy [53], are guideline-based blood tests for access to specialist care [44]. Brain natriuretic peptides are synthesised in astrocytes [54] and ventricles when the heart is subject to stretch (normally by increased volumes) [55]. Brain natriuretic peptides regulate natriuresis/diuresis and the prevention of fibrosis as well as hypertrophy of the heart [55].

### 2.3. Pneumonia

The relevance of the association between atypical antipsychotics and pneumonia and its lethality is well known [22]. The association between CLZ and pneumonia is also supported by numerous clinical studies [56,57,58,59]. A recent VigiBase search suggested that the relative lethality associated with pneumonia in CLZ (30%: 2077 fatal outcomes in 6983 cases) was approximately fifteen times higher than when associated with agranulocytosis [23] (Table.1). Indeed, a comparison search using VigiBase also reported that CLZ was particularly prone to developing pneumonia and had the highest lethality compared with other atypical antipsychotics, including risperidone, olanzapine, and quetiapine [23,60]. Therefore, pneumonia is one of the most severe lethal adverse CLZ reactions.

IThe elevation of plasma concentrations of CLZ during inflammation due to infection in patients who were chronically administrated and had stable plasma CLZ levels was demonstrated [61,62,63]. The major mechanisms of CLZ intoxication induced by inflammation were supported by preclinical findings that both the expression and activities of cytochromes P450 (CYP)1A2 and CYP3A4 [64] were reduced by some pro-inflammation cytokines, i.e., interleukin-1 (IL-1), IL-2, IL-4, IL-6, tumour necrosis factor (TNF)-α, TNF-β, interferon (IFN)-α, and IFN-γ [65]. Therefore, the lethality of pneumonia in CLZ patients was explained in that CLZ likely contributes to the pathomechanism of pneumonia, and the development of pneumonia can cause CLZ intoxication [66]. In other words, bidirectional interaction between pneumonia and CLZ intoxication is highly lethal.

### 2.4. Discontinuation Relapse Psychosis

The mainstay of therapy for CLZ-induced pneumonia, cardiotoxicity, and seizures is recommended CLZ cessation, specialist review, and instigation of disease modifying treatments [27,44,67]. Contrary to the benefit of the abrupt discontinuation of CLZ against several adverse CLZ reactions, there are a few mentions of the risk for the mental status of schizophrenia. The abrupt worldwide discontinuation of CLZ in 1975 by agranulocytosis resulted in 39% individuals who suffered a relapse of psychosis [68,69]. The relapsed psychosis associated with abrupt CLZ discontinuation was rapid-onset (3–14 days) and over five times more rapid in onset compared with those with haloperidol [70,71]. The reports regarding the unexpectedly high incidence of recurrent psychosis due to abrupt CLZ discontinuation were only available half a century ago, therefore, detailed investigations should be initiated.

## 3. Estimated Pathomechanisms of CLZ

### 3.1. Candidate Mechanisms of Clinical Efficicacy of CLZ on Treatment-Resistant Schizophrenia

It is well known that dopamine D2 receptor (D2R) antagonism with serotonin (5-HT) type 1A receptor (5-HT1AR) partial agonism or 5-HT type 2A receptor (5-HT2AR) antagonism contributed to the clinical features of an atypical antipsychotics class [72,73]. According to this criteria, the binding profiles of CLZ exhibited atypical antipsychotic properties due to the 5-HT1AR partial agonism and antagonism to D2R and 5-HT2AR [74,75]. The combination of receptor binding features of CLZ increased the extracellular levels of dopamine, 5-HT, and norepinephrine in the medial prefrontal cortex (mPFC), similar to other atypical antipsychotics, blonanserin, lurasidone, olanzapine, quetiapine, and zotepine [76,77,78,79,80,81]. Therefore, the effects of CLZ on monoaminergic receptors and transmission cannot fully explain the specific CLZ mechanism of superior efficacy against treatment-resistant schizophrenia.

The majority of atypical antipsychotics except for CLZ or aripiprazole did not affect γ-aminobutyrate (GABA) release in the mPFC, whereas both CLZ and aripiprazole reduced GABAergic transmission in the mPFC [76,77,78,79,80,81,82,83,84,85,86]. O’Connor and O’Shea investigated the sub-cortical dysfunction of GABAergic transmission that likely contributes to underlie the mechanisms of rapid-onset CLZ discontinuation-induced relapsed psychosis [87]. They demonstrated that the abrupt discontinuation of subchronic CLZ administration generated persistent, reduced GABA release in the ventral tegmental area (VTA), nucleus accumbens (NAc), and basal ganglia without affecting that in the mPFC [87].

Clinically, the functional abnormality of the glycine-sensitive site of N-methyl-D-aspartate/glutamate receptor (NMDAR) was identified in treatment-resistant schizophrenia [88,89,90]. Preclinically, CLZ (but not haloperidol) enhanced releases of L-glutamate, glycine, and D-serine resulting in the activation of NMDAR [86,91]. The stimulatory effects of CLZ on glutamatergic transmission likely contribute to the pathomechanism of effectiveness of CLZ for treatment-resistant schizophrenia and/or CLZ-induced seizures [92]; however, CLZ did not affect the neuronal resting membrane potential [93]. Taken together with clinical findings, these preclinical findings suggest that the enhanced NMDAR associated glutamatergic transmission induced by CLZ cannot be fully interpreted by neurotransmission alone, but can explain the mechanisms using tripartite synaptic transmission containing gliotransmission [76,77,84,86,94,95,96].

### 3.2. Impact of Tripartite Synaptic Transmission in Pathomechanisms of Neuropsychiatric Defeciency

Thalamocortical glutamatergic transmission has been identified as one of the bottom-up promoting system of cognition. The dysfunction of the integration of input signalling into the glutamatergic neurons in the mediodorsal thalamic nucleus (MDTN) contribute to the impairment of several types of cognition. Indeed, the deficit/disruption of MDTN exhibited deficits in the regulation of learning, memory, emotion, and perceptual integration [97,98,99,100,101]. Contrary, the tonic activation of the thalamocortical glutamatergic pathway in experimental animal models of schizophrenia, attention-deficit hyperactivity disorder, and autism has been observed [76,77,84,85,94,102,103,104,105,106,107].

**Figure 1:**
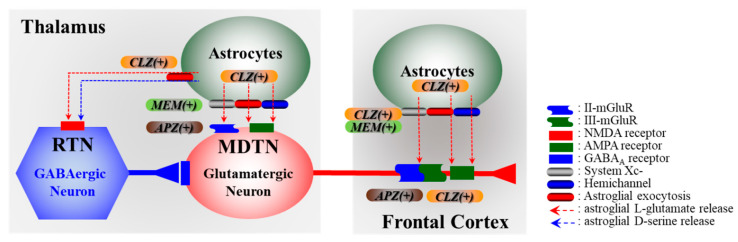
Effects of clozapine (CLZ), aripiprazole (APZ), and memantine (MEM) on glutamatergic transmission in the thalamocortical pathway from the mediodorsal thalamic nucleus (MDTN) to the frontal cortex. Glutamatergic neurons in the MDTN receive GABAergic inhibition from the reticular thalamic nucleus (RTN). Glutamatergic neurons in the MDTN also receive inhibitory and excitatory tripartite synaptic transmission via group II metabotropic glutamate receptors (II-mGluR) and glutamate/α-amino-3-hydroxy-5-methyl-4-isoxazolepropionic acid receptors (AMPAR), respectively. Astrocytes release L-glutamate through the transporter (system Xc-), hemichannel, and exocytosis mechanisms. Astrocytes also release D-serine via an activated astroglial exocytosis mechanism. The combination of astroglial-released L-glutamate and D-serine enhances the glutamate/N-methyl-D-aspartate receptor (NMDAR). L-glutamate output through system Xc- activates II-mGluR and III-mGluR in the thalamus and frontal cortex. The output of L-glutamate through the hemichannel activates AMPAR in the thalamus.

In particular, NMDAR-antagonist-induced tonic activation of thalamocortical glutamatergic transmission was compensated by modulation via the activation of the astroglial system Xc- and metabotropic glutamate receptors (mGluR) (Figure 1) [76,84,85,95,103,108,109]. CLZ supressed and enhanced thalamocortical glutamatergic transmission via the activation of group III mGluR (III-mGluR) and astroglial D-serine exocytosis in the frontal cortex (Figure 1) [84,86]. CLZ also enhanced thalamocortical glutamatergic transmission due to enhanced hemichannel activities in the frontal cortex and thalamus (Figure 1) [96].

Microdialysis studys demonstrated that CLZ alone weakly increased the release of L-glutamate in the frontal cortex, whereas CLZ surprisingly prevented NMDAR-antagonist (dizocilpine: MK801)-induced L-glutamate release [84,96]. During the resting stage, CLZ supressed MK801-induced thalamocortical glutamatergic transmission via the activation of III-mGluR in the frontal cortex [84], whereas after the activation of the hemichannel evoked by high K^+^ and removal Ca^2+^ stimulation, CLZ conversely enhanced the activated thalamocortical glutamatergic transmission in both the frontal cortex and thalamus (Figure 1) [96]. Additionally, the stimulatory effects of CLZ were enhanced time-dependently, since the activation of L-glutamate release induced by subchronic CLZ administration was larger than that by acute administration [96]. This time-dependent enhanced L-glutamate release induced by CLZ is caused by the increased connexin43 (Cx43) expression in the plasma membrane fraction [96]. These preclinical findings provide the important impact of the tripartite synaptic transmission in the double-edged sword clinical effects of CLZ, cognition promoting effects, and several adverse reactions.

## 4. Effects of CLZ on Cx43 and Its Associated Signal Transduction System

**Figure 2:**
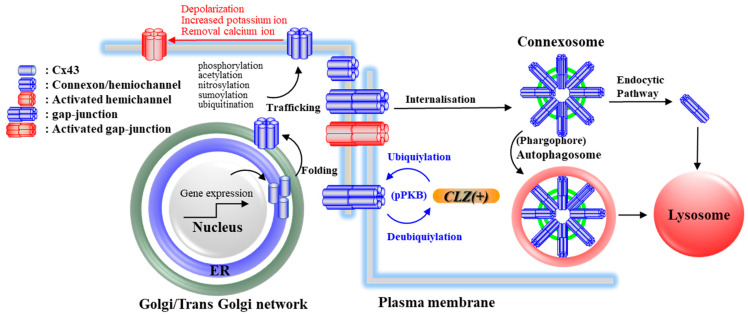
The life cycle of connexin43 (Cx43). Cx43 synthesis is regulated by several transcriptional factors (Sp1, activator protein 1 complex, cyclic AMP, and the Wnt pathway) and epigenetic processes (histone modifications, DNA methylation, and microRNA species). Synthesized six Cx43 fold oligomerise to form a connexon in the endoplasmic reticulum (ER) and trans-Golgi network. Trafficked connexons to the plasma membrane become hemichannels as functional connexons, and dock with connexons on neighbouring cells to form gap-junctions. The trafficking process of connexons is regulated by various signalling systems, including phosphorylation, acetylation, nitrosylation, sumoylation, and ubiquitination. CLZ likely activates connexon trafficking via enhanced ubiquitylation via the phosphorylation of protein kinase B (pPKB). Both hemichannels and gap-junctions are low open probabilities during the resting stage; however, the elevation of extracellular potassium ions, the removal of extracellular calcium ions, or the depolarization activation of the hemichannel/gap-junction leading to the persistent opening of Cx43 containing channels. The internalisation of the gap-junction generates the connexosome (also known as an annular gap-junction). The connexosome is degraded by autophagy via the formation of an autophagosome or endocytic pathway. The Cx43 turn-over is very fast in the brain, and the half-life is several hours.

Connexin (Cx) is a family of 21 protein isoforms [110,111,112]. Six Cx units assemble to form homomeric or heteromeric connexons (Figure 2). Two connexons in two neighbouring cells form a gap-junction channel with an aqueous pore and charged surface walls (Figure 2) [110,111,112], whereas a single connexon contributes to a chemical connection between intra- and extracellular spaces as a hemichannel [110,111,112] (Figure 2). Contrary to physiological conditions, pathological hyperactivated conditions generate persistent hemichannel/gap-junction openings, which lead to disrupting several homeostasis systems [110,111]. Cx43 is the most widely and predominant expressed Cx subtype, in the astroglial, myocardial, and pulmonary gap-junction/hemichannel Cx [113,114].

The transcription of Cx43 is regulated by several transcriptional factors (activator protein 1 complex (Sp1), cyclic adenosine monophosphate (cAMP), and the wingless (Wnt) pathway) and epigenetic processes (histone modifications, DNA methylation, and microRNA species) [113,115]. Histone deacetylase (HDAC) inhibitors increased the acetylation of histone and non-histone proteins leading to activated transcription, enhanced gene expression, and modification of the function of target proteins [116]. We demonstrated that HDAC inhibitors, (suberoylanilide hydroxamic acid, 4-phenylbutyrate and trichostatin A) increased the expression of Cx43 mRNA and protein [117,118,119]. HDAC inhibition is considered to be one of the most principal pharmacological targets of valproate (VPA), and inhibits class I and IIa HDAC isoforms [116]. VPA increased the astroglial Cx43 expression in the cytosol without affecting that in the plasma membrane via HDAC inhibition [96].

The post-translational modification of Cx43, including the phosphorylation, acetylation, nitrosylation, sumoylation, and ubiquitylation play important roles in the Cx function and expression in the plasma membrane (folding, trafficking, accretion, docking, and degradation) [113]. The phosphorylation of Cx43 was found to be regulated by the action of more than ten kinases and phosphatases, including mitogen-activated protein kinase (MAPK)/extracellular signal-regulated kinase (Erk) signalling (Figure 2) [120,121]. The role of the ubiquitylation of Cx43 is considered to be regulated by protein kinase B (PKB) (Figure 2) [122,123]. CLZ increased the astroglial Cx43 expression via post-translational modification rather than transcription, as CLZ increased the Cx43 expression in the plasma membrane fraction higher than that in the cytosol fraction [96].

Unfortunately, the mechanism of the stimulatory effects of CLZ on the process of Cx43 expression in the plasma membrane via post-translational modification system remains to be clarified. Among the revealed phosphorylation/ubiquitylation processes of Cx43, the activation of PKB signalling induced by CLZ [19,124] provides a notable molecule underling the mechanisms of Cx43 expression and impaired glucose tolerance [125,126]. CLZ increased the time- and concentration-dependently increased Ser473 phosphorylated-PKB (pPKB) [127,128]. An activated pPKB increased Cx43 expression [129], whereas PKB knockout mice decreased the Cx43 expression, which preceded heart contractile dysfunction [130]. These preclinical findings suggest that CLZ increased the Cx43 expression in the plasma membrane via the activation of pPKB in several organs.

## 5. Candidate Pathomechanisms of CLZ Associated with Cx43

### 5.1. Cognition and Cx43

Although we have no evidence indicating any abnormalities of molecules associated with gap-junctions or hemichannels in the brains or genomes of individuals with schizophrenia, the accumulated findings suggest that the functional abnormalities of gap-junctions and hemichannels lead to severe cognitive impairment in schizophrenia [131,132] due to disorganization in neuro-glial networks, causing a neurotransmission imbalance in specific brain regions. Therefore, the dysfunction of gap-junctions and hemichannels plays key roles in the pathophysiology but not pathogenesis of schizophrenia as a possible reversible functional abnormality that is able to be compensated for by therapeutic intervention.

**Figure 3:**
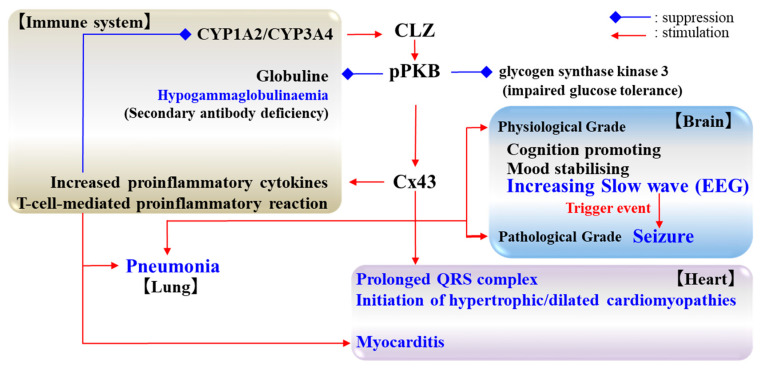
The proposed hypothesis of the pathomechanisms of clinical efficacy and severe adverse reactions of CLZ. Electroencephalogram (EEG).

CLZ was the first atypical antipsychotic showing cognitive promoting functions in patients with schizophrenia [133,134]. A recent systematic review and meta-analysis study demonstrated that the efficacy of CLZ was almost equal to other antipsychotics in manic episodes, but was superior to other antipsychotics against treatment-resistant bipolar disorder [135]. The clinical prognosis of treatment-resistant bipolar disorder is considered to be heterogeneous and not a general rule [136,137], therefore, improvement of cognitive impairment is an important outcome [135]. A model of imbalances in the tripartite synapses responsible for the pathophysiology of bipolar disorder has been proposed [138,139]. This hypothesis emphasizes that the downregulation of astroglial Cx contributes to the pathophysiology of depressive mood cognitive impairment [138,139]. Therefore, the upregulation of astroglial Cx43, and its associated gliotransmission, plays important roles in the mood stabilizing and cognitive promoting action of CLZ (Figure 3).

Cx43 turnover is very fast, and its half-life is several hours [140]. The manifestation of high incidence and rapid-onset of recurrent/relapse psychosis due to CLZ discontinuation was shown [68,70,71]. The extremely fast turn-over of Cx43 might be involved in the clinical features of recurrent/relapse psychosis due to CLZ discontinuation.

### 5.2. Seizure and Cx43

Clinically, Cx43 expression was upregulated in the patients with anticonvulsant-resistant focal epilepsy, including focal cortical dysplasia and temporal lobe epilepsy [141,142,143]. Numerous studies reported that the development of epilepsy upregulated Cx43 expression, of which, the intensity of Cx43 expression was affected by the epileptic seizures [141,144,145]. Research suggested that the upregulation of Cx43 in astrocytes exacerbated epileptic seizures in patients with temporal lobe epilepsy [143]. Preclinically, the upregulation of Cx43 in the focus region and focus generating neural circuits of a genetic sleep-related hypermotor epilepsy model, which included carbamazepine-resistant/zonisamide-sensitive epileptic seizures, were also observed [105,106]. Notably, both inputs of physiological and pathological neuronal excitability in the Cx43 upregulation regions provided the generation of epileptic discharges and accelerated the propagation of epileptic hyperexcitability [103,104,105,106,108]. Indeed, zonisamide and lacosamide, which clinically suppressed carbamazepine-resistant epileptic seizure of sleep-related hypermotor epilepsy, reduced Cx43 expression and its function, respectively [103,105,106], whereas the anticonvulsive target of carbamazepine in astrocytes was not Cx43 [103,146].

Taken together with both the clinical and preclinical findings, the combination between the stimulatory effects of CLZ on Cx43 expression in astroglial plasma membranes and L-glutamate output led to enhanced seizure susceptibility (Figure 3). The lack of acute VPA administration on astroglial transmission during chronic CLZ administration can explain the mechanisms of efficacy and safe augmentation therapy of CLZ due to less cognitive impairment induced by Cx43 [147]. Interestingly, during the chronic administration of CLZ, adjuvant acute VPA administration did not affect astroglial L-glutamate release, whereas the chronic administration of VPA drastically increased CLZ-induced astroglial L-glutamate release [96]. Similar to L-glutamate release, during chronic CLZ administration, VPA acutely did not markedly affect the Cx43 expression in the plasma membrane fraction, whereas during chronic VPA administration, CLZ acutely increased the Cx43 expression in the plasma membrane fraction [96]. These time-dependent interactions between CLZ and VPA on the Cx43 expression can provide the mechanisms of clinical efficacy and adverse reactions of CLZ.

### 5.3. Cardiotoxicity and Cx43

Normally, Cx40 (the atrial myocytes, in the atrioventricular node, His-bundle, and the ventricular conduction system), Cx43 (atrial and ventricular myocytes) and Cx45 (sinoatrial node and the atrioventricular node) are expressed in the heart [148,149]. Gap-junctions, which interact with adherens junctions, localize to the intercalated disks, in discrete regions of cardiomyocyte–cardiomyocyte coupling in the heart [150]. This system regulates the right maintenance of the cardiac rhythm, the regulation of vascular tone and endothelial function, as well the metabolic interchange between adjacent cells [151]. A reduction of 90% of Cx43 expression led to a 50% decrease in the conduction velocity, whereas a 50% reduction in Cx43 displayed some conduction slowing, and high levels of electrical uncoupling were needed to increase the arrhythmogenicity [152,153].

In contrast, the upregulation of Cx43 generated the prolongation of the QRS complex duration (Figure 3) [154]. Therefore, abnormalities of quantity, phosphorylation, or the distribution of Cx43 expression led to cardiac electrically conductive arrhythmia, as Cx43 remodelling generated ischemic arrhythmia via an overload of the intercellular Ca^2+^ [155]. The upregulation of Cx43 expression was increased in the initiation of hypertrophic and dilated cardiomyopathies, but was decreased with their progression into heart failure [156,157,158]. Therefore, the upregulation of Cx43 in the acute stages of hypertrophic and dilated cardiomyopathies may act as the trigger of pathological or pathophysiological onset (Figure 3). Another model indicated the dispersion of Cx43 over the entire cell surface and a proportional decrease in Cx43 at the intercalated disc centres [159,160]. These clinical and preclinical findings suggest that the abnormality of Cx43 expression contributes to functional and morphological dysfunction of the heart.

The association of risk of CLZ-induced myocarditis/cardiomyopathies with rapid titration or VPA administration at the commencement of CLZ [50] are explained in that the increased Cx43 in cytosol in cardiac cells is drastically trafficked to the plasma membrane resulting in the toxic hyperfunction of Cx43-associated hemichannels and gap-junctions.

### 5.4. Pneumonia and Cx43

Pneumonia is an inflammatory condition of the lungs, commonly caused by infection and less commonly by other numerous functional abnormalities including immune-functional deficiencies and certain medications [161]. Both clinical and preclinical studies demonstrated that upregulated gap-junction/hemichannel containing Cx43 and Cx40 in T-lymphocytes were involved in non-infectious pulmonary inflammation [162,163,164]. Additionally, the inhibition of the expression and/or function of Cx (Figure 3) [162,163,164] improved the proinflammatory reaction via decreasing the proportion of CD4+ T-lymphocytes with the levels of proinflammatory cytokines [162,163,164,165,166].

Carbenoxolone, a Cx gap-junction/hemichannel inhibitor [103,105,106], ameliorated pulmonary inflammation in asthma animal models via the possible reduction in interleukin-4 and -5 production and decreasing the infiltration of inflammatory cells in perivascular regions (Figure 3) [167]. In addition, carbenoxolone also decreased the differentiation of Th17 cells by a reduction in interleukin-23 production in antigen presenting cells [168]. A recent preclinical study using pulmonary hypertension/inflammation model rats demonstrated that carbenoxolone ameliorated various abnormalities of both pulmonary hypertension and inflammation, including right ventricular hypertrophy, pulmonary arteriolar remodelling, lung fibrosis, inflammatory cell infiltration, pulmonary arterial wall thickening, collagen deposition, pro-inflammatory cytokine production, and CD3+ and CD4+ T-lymphocytes accumulation in lung tissues via the inhibition of Cx43 upregulation in CD4+ and CD8+ T-lymphocytes in lung tissues [169].

CLZ-associated secondary antibody deficiency was only recently described [170,171,172,173]. CLZ-associated hypogammaglobulinemia was recently described as a treatable cause of sinopulmonary infection susceptibility [170,171,172,173]. A model disease for elucidating the mechanism of CLZ-induced secondary immune deficiency syndrome was already identified. The hyperfunction of phosphoinositide 3-kinase (PI3K) signalling elevated the phosphorylation of PKB resulting in primary antibody deficiencies, namely activated PI3Kδ syndrome (APDS) [174,175,176]. The blood analysis of patients with APDS revealed that naive T cell and memory B cell counts were reduced, and T cell blasts displayed enhanced activation-induced cell death, which was corrected by the addition of the PI3Kδ inhibitor [175]. The above findings suggest that the stimulatory effects of CLZ on PI3K/PKB signalling contributed to the development of pathomechanisms of severe pneumonia due to a complex immunodeficiency and inflammatory reaction through hyperactivated pPKB (Figure 3).

## 6. Remaining Challenges and Conclusions

In this review, based on the accumulating clinical and preclinical findings of CLZ, we sought to manifest a novel pathophysiology of clinical efficacy to treatment-resistant schizophrenia and the severe adverse reactions of CLZ. The receptor binding profile of CLZ could not fully provide the mechanisms of superiority of CLZ compared with other atypical antipsychotics for treatment-resistant schizophrenia. Research suggested the possibility that the enhancement of tripartite synaptic transmission associated with Cx43 induced by CLZ plays important roles in the mechanisms of clinical efficacy to treatment-resistant schizophrenia of CLZ. The upregulation/hyperactivation of Cx43, which is a predominantly expressed hemichannel/gap-junction constructive molecule in the brain, heart, and lungs, could also show a consistent mechanism regarding adverse reactions of CLZ. The upregulated Cx43 likely contributes to following several double-edged clinical actions of CLZ.

(1) Cognitive promoting and mood stabilization.
(2) Hyperactivation of excitatory tripartite synaptic transmission.
(3) Dysfunction of the immune-responses in the heart and lungs.
(4) Dysfunction of the propagation of the electrical impulses in the heart.

The enhanced Cx43 expression in the plasma membrane induced by CLZ is modulated by various post-translational regulation systems, including phosphorylation, acetylation, nitrosylation, sumoylation, and ubiquitylation, but possibly is not modulated by the transcriptional system. Until recently, the mechanisms of the stimulatory effects of CLZ on Cx43 expression in the plasma membrane remained to be clarified; however, the accumulating pharmacodynamic findings of CLZ to date suggest that CLZ possibly activates the phosphorylation of PKB resulting in the enhanced ubiquitylation of Cx43.

The detailed mechanisms of the effects of CLZ on PKB activity must be clarified preclinically. Particularly, PKB activation was suggested to contribute to CLZ-associated secondary antibody deficiency/hypogammaglobulinemia (see detailed Section 5.4) and impaired glucose tolerance [125,126]. The phosphorylation of PKB/Akt2 inhibited the activity of glycogen synthase kinase 3 resulting in an increase in glycogen synthesis [177]. A preclinical study demonstrated that CLZ also inhibited the activity of glycogen synthase kinase 3 (Figure 3) [178].

Therefore, although in this review, we emphasized the importance of Cx43 upregulation regarding the double-edged clinical action of CLZ, PKB activation is likely a more fundamental molecule for the clinical action of CLZ. Searching for biomarkers available in blood samples could be a viable project if PKB activation plays an important role in the mechanism of clinical efficacy and severe adverse reactions of CLZ. If the mechanism of peripheral severe adverse reactions is the same as that of the clinical efficacy of CLZ, medication, which lacks blood brain barrier transit, avoiding abrupt CLZ discontinuation is sufficient for the treatment of peripheral adverse CLZ reactions.

## Figures and Tables

**Table 1:** Summary of the major adverse reactions in the blood, lung, heart, and brain associated with clozapine (CLZ) of reported cases, fetal outcomes and relative lethality rate (%) in the World Health Organization’s global database (VigiBase) [23].

Adverse Reaction	Cases	Fatal Outcomes	Relative Lethality (%)
Agranulocytosis	34,931	550	1.6
Pneumonia	6983	2077	29.7
Arrhythmia	6927	319	4.6
Myocarditis	4586	539	11.8
Sudden death/Cardiac arrests	1614	1449	89.8
Cardiomyopathy	1132	131	11.6
Seizure	6231	308	4.9

## Abbreviations

5-HT	serotonin
5-HT1AT	serotonin receptor type 1A
5-HT2AR	serotonin receptor type 2A
cAMP	cyclic adenosine monophosphate
CLZ	clozapine
Cx	connexin
Cx43	connexin43
CYP	cytochromes P450
D2R	dopamine D2 receptor
ECG	electrocardiogram
EEG	electroencephalograph
ER	endoplasmic reticulum
Erk	extracellular Signal-regulated Kinase
FDA	the Food and Drug Administration
FIN11	11-year follow-up of mortality in patients with schizophrenia: a population-based cohort
GABA	γ-aminobutyrate
HDAC	histone deacetylase
IFN	interferon
IL	interleukin
MAPK	mitogen-activated protein kinase
mPFC	medial prefrontal cortex
NAc	nucleus accumbens
NMDAR	N-methyl-D-aspartate/glutamate receptor
PI3K	phosphoinositide 3-kinase
PKB	protein kinase B
pPKB	phosphorylated protein kinase B
Sp1	activator protein 1 complex
SSRI	selective serotonin reuptake inhibitor
TNF	tumour necrosis factor
VigiBase	the World Health Organization global database
VPA	valproate
VTA	ventral tegmental area
WHO	the World Health Organization
Wnt	wingless
